# Safety and efficacy of rapid withdrawal of antiseizure medications during long‐term video‐electroencephalogram monitoring in children with drug‐resistant epilepsy: A retrospective study

**DOI:** 10.1002/epi4.12680

**Published:** 2023-02-17

**Authors:** Shuang Wang, Wen Wang, Guojing Yu, Lin Wan, Yuying Fan, Hongjie Wang, Tong Liu, Taoyun Ji, Qingzhu Liu, Lixin Cai, Xiaoyan Liu

**Affiliations:** ^1^ Pediatric Epilepsy Center Peking University First Hospital Beijing China; ^2^ Department of Pediatrics Peking University First Hospital Beijing China; ^3^ Senior Department of Pediatrics The Seventh Medical Center of PLA General Hospital Beijing China; ^4^ Department of Pediatrics, The First Medical Center Chinese PLA General Hospital Beijing China; ^5^ Department of Pediatrics Shengjing Hospital of China Medical University Shenyang China

**Keywords:** children, epilepsy, long‐term video‐electroencephalographic monitoring, protocol, withdrawal of antiseizure medications

## Abstract

**Objective:**

Performing long‐term video‐electroencephalographic monitoring (LTVEM) to obtain the ictal electroencephalogram (EEG) is important for presurgical evaluation. This study aimed at investigating the safety and efficacy of our protocol developed at Peking University First Hospital (PUFH) for rapid withdrawal of antiseizure medications (ASMs) during LTVEM to induce seizures in children with drug‐resistant epilepsy (DRE) exhibiting nondaily seizures.

**Methods:**

Children with DRE who followed the PUFH protocol for rapid withdrawal of ASMs during LTVEM between 2018 and 2021 were enrolled. The occurrence of seizures, number of ASMs withdrawn, seizure onset time after ASM tapering initiation, changes in interictal epileptiform discharge (IED), and adverse events were evaluated during LTVEM.

**Results:**

Among 80 children evaluated in this study, seizures were induced successfully in 72 (90%) children. Furthermore, no change in IED sites was observed in these 72 children following the initiation of ASM tapering while 2 children exhibited secondary bilateral tonic‐clonic seizures. The median time from ASM tapering initiation to the onset of the first seizure was found to be 3 days (2–4), while the median number of ASMs withdrawn was 2 (1–2). Finally, 66 children (91.7%) had habitual seizures while 6 children had nonhabitual seizure semiology.

**Significance:**

The PUFH protocol can be used for the rapid withdrawal of ASMs during LTVEM in children with DRE. Using this protocol, ictal EEG patterns can be obtained in a relatively short time for most patients with fewer adverse effects during LTVEM, which may provide meaningful electro‐clinical information for presurgical evaluation.

**Key Points**

- One ASM withdrawal per day during LTVEM is acceptable.
- By the taper of one or two ASMs, it is possible to induce a habitual seizure within 4 days in most children with DRE.
- Protocol of PUFH during LTVEM provides meaningful information for presurgical evaluation.

## 1 INTRODUCTION

Epilepsy is a neurological disorder that affects people of all ages. Although several antiseizure medications (ASMs) are available that can control seizures, in nearly one‐third of the patients, the disease progresses to drug‐resistant epilepsy (DRE).^1^ It has been shown that vagus nerve stimulation, ketogenic diet, and other treatments exert beneficial effects in patients with DRE.^2^, ^3^ However, these treatment modalities failed to provide seizure freedom in the majority of patients, and resective surgery remains the ultimate treatment option for patients with DRE.^4^

The success of epilepsy surgery depends on the accurate localization of the epileptogenic zone (EZ) during presurgical evaluation. Capturing seizures, especially habitual seizures during long‐term video‐electroencephalographic monitoring (LTVEM), is essential for presurgical evaluation.^5^ Although there is a certain level of periodicity, seizures are mostly unpredictable, and the duration between seizures can vary in these patients. In some patients, the duration between two seizures can be more than 1 week, while in others, it can be even 1 month. Therefore, the ictal EEG cannot be obtained even while performing LTVEM,^6^ which can provide meaningful electro‐clinical information of EZ localization for epilepsy surgery.

Currently, ASM withdrawal is the preferred method to obtain ictal EEG in epilepsy patients that have longer intervals between seizures.^5^, ^7^, ^8^ However, ASM withdrawal protocols during LTVEM vary among epilepsy centers.^9^, ^10^, ^11^, ^12^, ^13^ Based on the published evidence mainly from systematic reviews and meta‐analysis studies, the International League Against Epilepsy (ILAE) has published a standard guideline for LTVEM, which strongly recommends that in patients without a history of status epilepticus (SE) or frequent daily seizures, a fast taper of 30%–50% daily ASM dosage should be considered.^6^ However, the study population in these previous studies has a wide age distribution and included adults as well as children, and there are no protocols for ASM tapering specifically for children.^6^ Herein, we retrospectively analyzed the outcomes of our protocol for rapid withdrawal of ASMs to induce seizures during LTVEM at the Pediatric Epilepsy Center of Peking University First Hospital (PUFH). We also evaluated the efficacy and safety of this protocol to provide our own experience to other pediatric neurologists for reference.

## 2 METHODS

### 2.1 Subjects

Children with epilepsy who underwent LTVEM during the period from January 2018 to December 2021 at PUFH for presurgical evaluation were enrolled.

The inclusion criteria were (i) patients under 18 years of age requiring presurgical assessment with the definitive diagnosis of DRE^14^ and (ii) patients having nondaily seizures prior to admission (based on the disease history and EEG obtained on the first day). The exclusion criteria were (i) patients on ASM (regardless of dose or type) started within 2 weeks prior to admission, (ii) patients who did not follow our protocol for ASM withdrawal (eg, ASM withdrawn by the caregivers of children on their own or withdrawn without proper dose adjustment), and (iii) patients with no seizures during LTVEM and the monitoring period was less than 7 days due to intolerance.

Demographic and clinical data were recorded for all patients, including seizure types before and after ASM tapering initiation, types and doses of ASMs before and during LTVEM, and seizure onset time during ASM tapering.

Since this was a retrospective cohort study, the identity of all patients remained anonymous. The requirement for informed consent was waived by the Ethics Committee of Peking University First Hospital due to the observational nature of the study.

### 2.2 LTVEM

All patients were monitored using the 32‐channel Nihon Kohden EEG‐1200C video EEG system. According to the international 10–20 system, 19 channels with subzygomatic T1 and T2 electrodes were selected. Electrocardiogram and electromyography of the bilateral deltoid and quadriceps femoris were recorded simultaneously, and bilateral forearm, eyelid, or perioral electrodes were added according to the semiology before LTVEM. The monitoring lasted until the seizure was induced or for 7 days of total monitoring even without the induction of seizures.

### 2.3 ASM withdrawal protocol during LTVEM

#### 2.3.1 Indications for ASM tapering

If no seizure was detected during the first 24‐hour EEG monitoring, ASM tapering was deemed necessary.

#### 2.3.2 ASM selection for withdrawal

The medicine was considered effective if no seizure occurred during the medication or the seizure frequency was reduced by at least 50%. Based on the disease history and the effective duration of the treatment, ASMs were selected for withdrawal. When the drug was effective but the duration of treatment was shorter than that of any previously used drugs, the newly added ASM was first withdrawn.

#### 2.3.3 ASM withdrawal

After the first 24‐hour EEG monitoring with no seizure, ASM tapering began in a certain order. The dose of ASM that has to be withdrawn first was reduced to half every 12 hours. If there was no seizure after the second 24‐hour EEG monitoring, a similar protocol was used with the second ASM that has to be withdrawn. If there was still no seizure, all ASMs were withdrawn. On the last monitoring day, all ASMs were restored to the original dose.

### 2.4 Evaluation of IED

EEG data of the entire monitoring period were interpreted by two experienced senior pediatric electrophysiologists (Wang W and Yu GJ). The IED site before the initiation of ASM tapering was identified and defined as the baseline site. If new IED sites appeared after withdrawal, the increment in the IED site was determined. If the original IED site disappeared after withdrawal, the disappearance of the baseline site for IED was determined.

### 2.5 Evaluation of seizures

Seizure events were identified by guardians after analyzing the video recorded during LTVEM. Seizures induced following ASMs tapering were categorized as habitual seizures if they were similar to those observed before LTVEM or new seizure types if they were different.

### 2.6 Assessment of serious adverse events

Serious adverse events (SAEs) were defined as the occurrence of the secondary bilateral tonic‐clonic seizure (BTCS), seizure clusters, status epilepticus (SE), cardiac arrhythmia, the requirement of rescue treatment with midazolam, postictal psychosis, musculoskeletal injuries, and scalp abrasion.^10^

### 2.7 Data analysis

Statistical analysis was performed using the SPSS 26.0 software. Data are expressed as mean ± SD for normally distributed variables or median (interquartile range) for nonnormally distributed variables. Kaplan‐Meier (KM) survival curves were plotted for analyzing seizure events.

## 3 RESULTS

**FIGURE 1:**
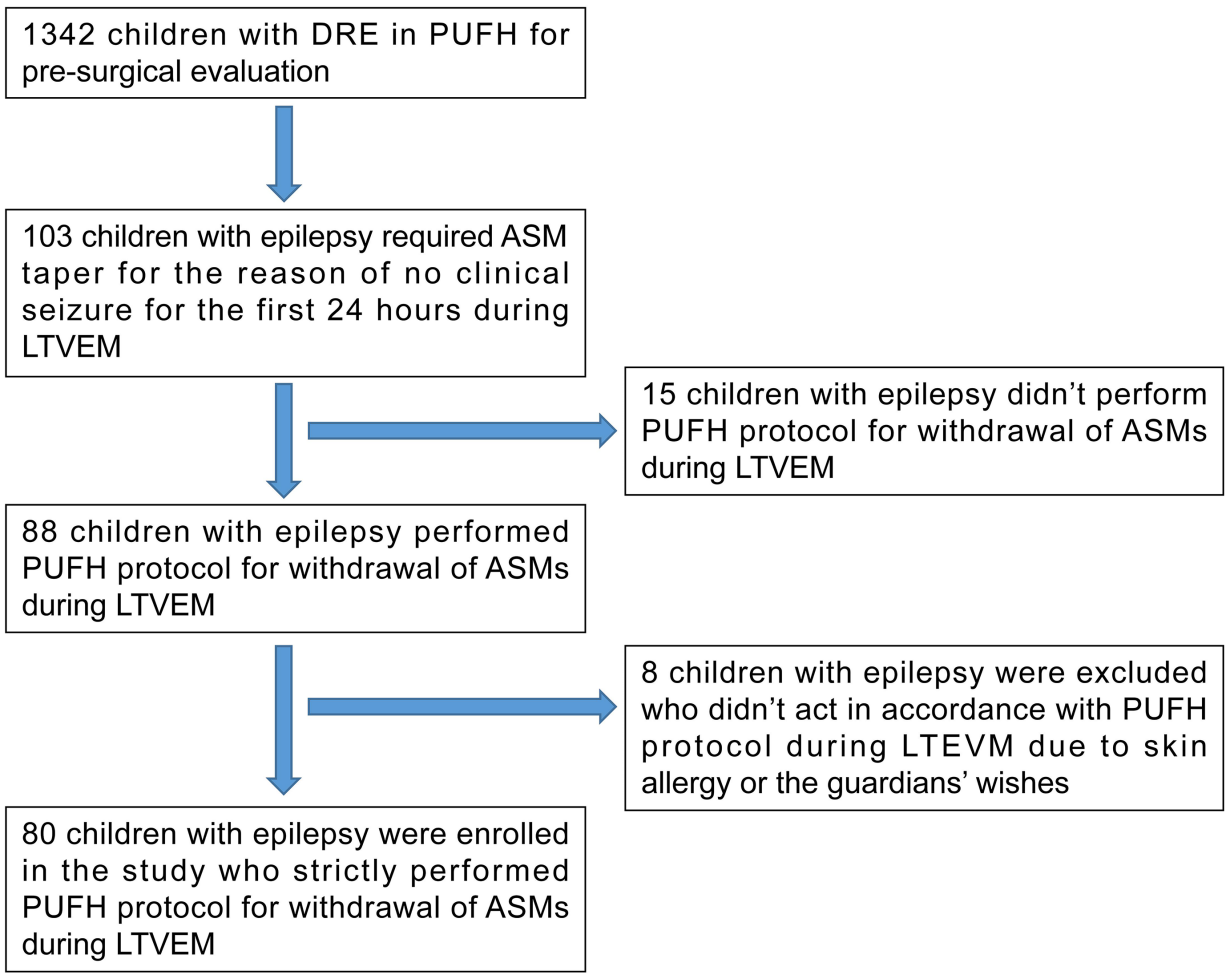
Study design. DRE, drug‐resistant epilepsy; ASM, antiseizure medication

The data of 1342 children with DRE who underwent LTVEM for presurgical evaluation at our epilepsy center from January 2018 to December 2021 were analyzed. Among them, 103 children had no seizures during the first 24‐hour monitoring period and required the PUFH protocol for rapid ASM withdrawal. In 15 children, ASM was withdrawn by the guardians on their own or without proper dosage adjustment; hence, they were excluded. Furthermore, 8 children who had no seizures during LTVEM and the monitoring time was <7 days due to other reasons, including the unwillingness of their guardians and skin allergy, were also excluded from the study. Finally, the data of 80 patients who followed the PUFH protocol strictly were analyzed (Figure 1). Demographic and clinical data were collected, and ASMs that were used in more than 10% of all the patients in this cohort are listed in Table 1.

**TABLE 1:** Demographic data of patients included in our study

	Patients who underwent LTVEM (*n* = 80)	
Gender		
Male	44 (55%)	
Female	36 (45%)	
Age at EEG monitoring (months) (median, range)	32.5 (13, 214)	
Seizure frequency		
Weekly	46 (57.50%)	
Monthly	29 (36.25%)	
Yearly	5 (6.25%)	
ASMs exposure		
OXC	51 (63.75%)	
VPA	42 (52.50%)	
LEV	39 (48.75%)	
LTG	19 (23.75%)	
TPM	19 (23.75%)	
LCM	11 (13.75%)	
Number of ASMs used		
5	2 (2.50%)	
4	9 (11.25%)	
3	30 (37.50%)	
2	32 (40%)	
1	7 (8.75%)	
Seizure onset during LTVEM		
Yes	72 (90%)	
No	8 (10%)	
	All patient with LTVEM(*n* = 80)	Patients experienced seizure onset at LTVEM (*n* = 72)
Duration between seizure onset and ASM withdrawal (days)		
1	11 (13.75%)	15.28%
2	23 (28.75%)	31.94%
3	15 (18.75%)	20.83%
4	14 (17.50%)	19.44%
5	6 (7.50%)	8.33%
6	3 (3.75%)	4.17%
Number of ASMs withdrawn at seizure onset		
1	31 (38.75%)	43.06%
2	32 (40%)	44.44%
3	8 (10%)	11.11%
4	1 (1.25%)	1.39%

Abbreviations: ASM, antiseizure medication; EEG, electroencephalography; LCM, lacosamide; LEV, levetiracetam; LTG, lamotrigine; LTVEM, long‐term video‐electroencephalographic monitoring; OXC, oxcarbazepine; VPA, valproic acid.

**FIGURE 2:**
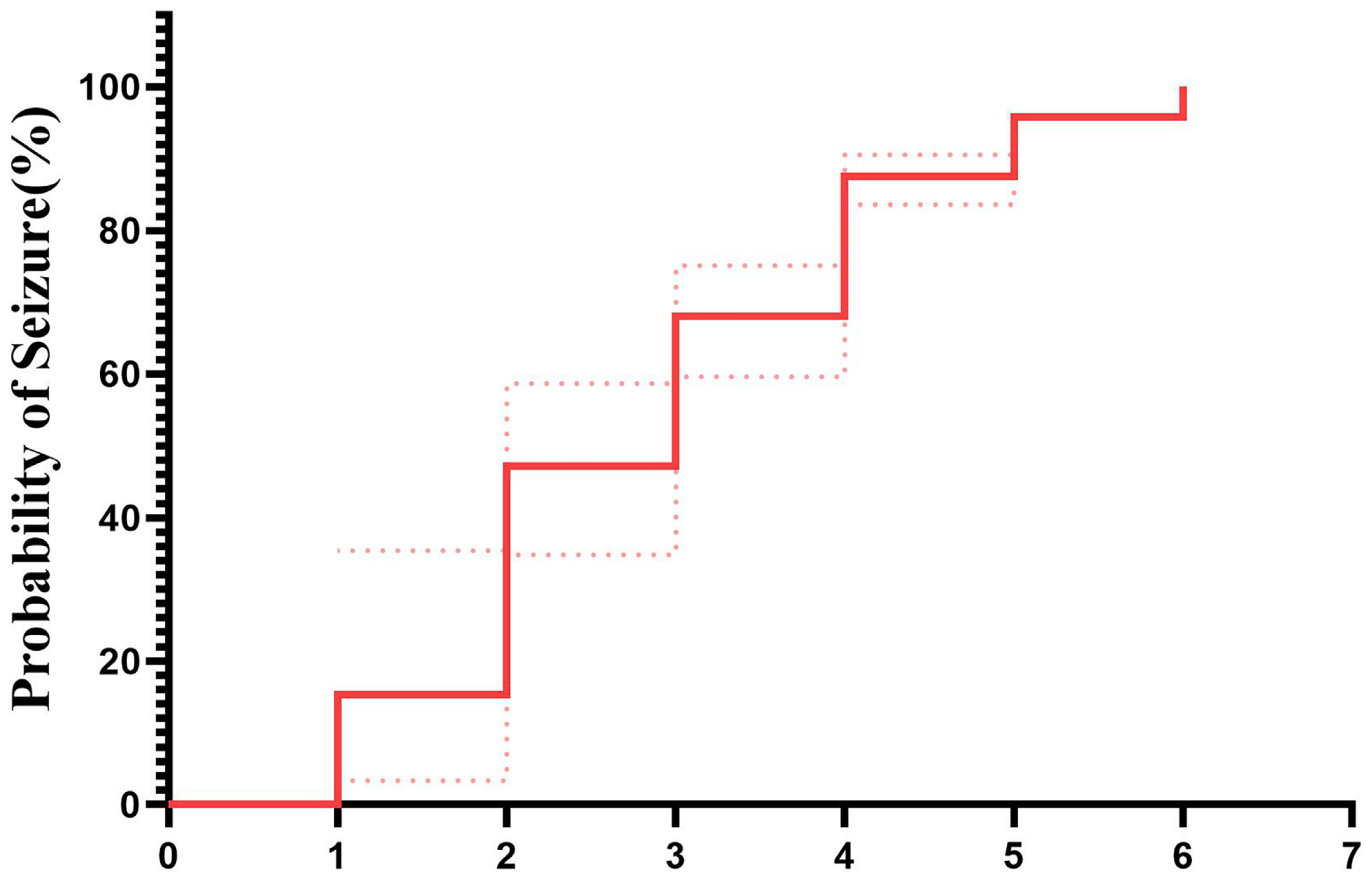
Time curve showing the relationship between seizure events and the duration following ASM withdrawal in 80 patients. Dotted line: 95% confidence interval. ASMs, antiseizure medications.

**FIGURE 3:**
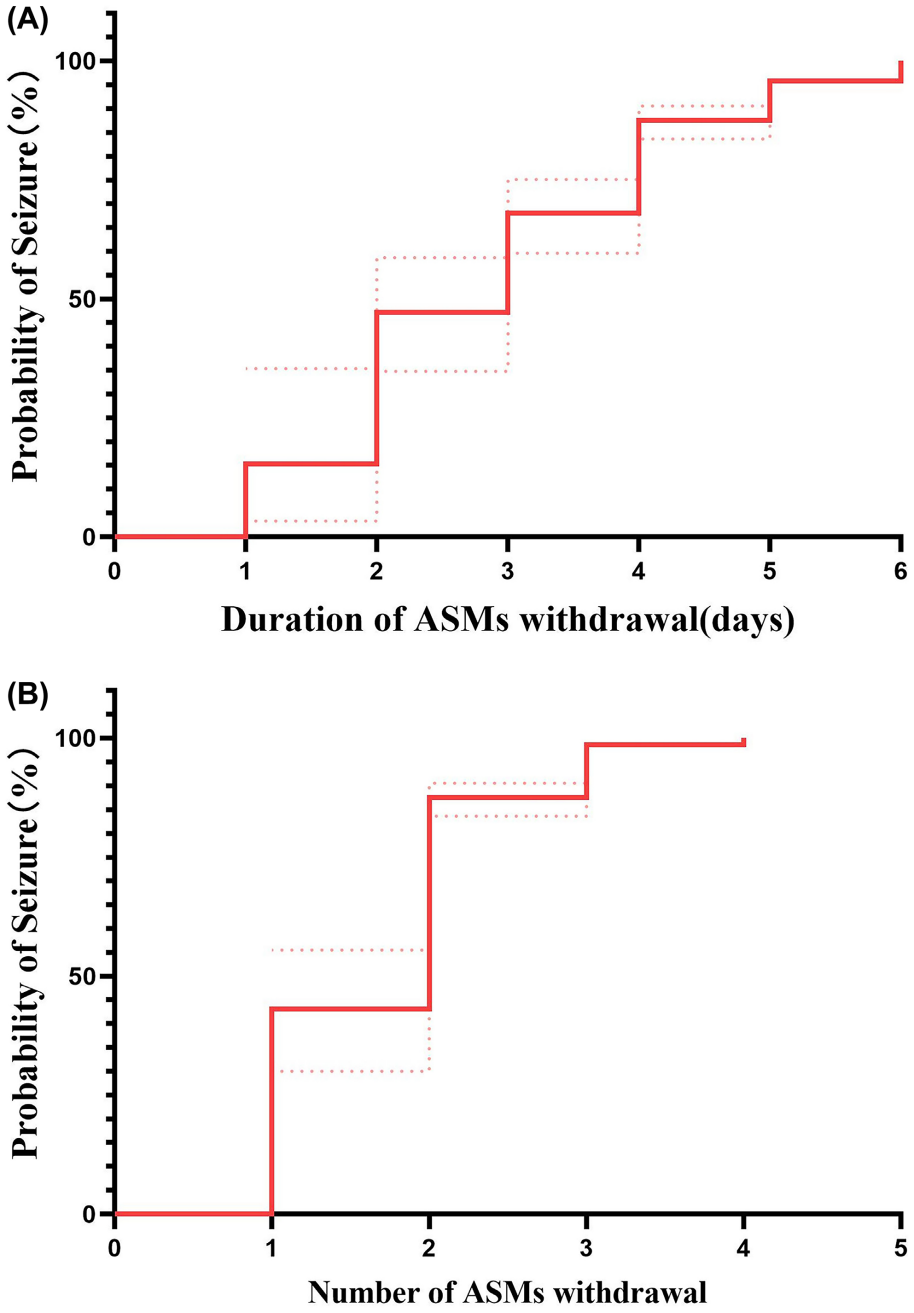
Time curve showing the relationship between seizure events and the duration following ASM withdrawal as well as the number of ASMs withdrawn in 72 epileptic children in whom seizures were induced successfully. (A) Time curve showing the relationship between seizure events and the duration following ASM withdrawal. (B) Time curve showing the relationship between seizure events and the number of ASM withdrawn. Dotted line: 95% confidence interval. ASMs, antiseizure medicines.

Of the 80 patients, 44 were males and 36 were females, with a median age of 32.5 (1st quartile–3rd quartile: 11, 70.25) months. The median number of ASMs taken by the patients before LTVEM was 3 (1st quartile–3rd quartile: 2–3). Furthermore, in 72 (90%) patients, seizures were induced successfully, with 63 (79.9%) patients exhibiting seizures within 4 days after the initiation of ASM tapering. Moreover, 8 (10%) patients had no seizures during the 7‐day monitoring period after the withdrawal of all ASMs (Figure 2). Among 72 children in whom seizures were induced successfully, the median time from ASM tapering initiation to the onset of the first seizure was 3 days (1st quartile–3rd quartile: 2–4) (Figure 3A). and median number of ASM before LTVEM was 3 (1st quartile–3rd quartile: 2–3). The median number of ASMs withdrawn during LTVEM was 2 (1–2) (Figure 3B).

Of the 80 children included in our study, 8 (10%) developed new IED sites or had generalized IED, and none showed disappearance or decrease in the number of original IED sites. Furthermore, of the 72 children with successful induced seizures, 66 (91.7%) had habitual seizures while 6 (8.3%) had new seizure types with no change in IED sites. Besides 2 patients who experienced BTCS, no other patient had SAEs.

## 4 DISCUSSION

The acquisition of ictal EEG during LTVEM is one of the most important steps in the presurgical evaluation of patients with DRE. Because of the low cost and benefits of LTVEM, it is the commonly used method to induce seizures in patients with epilepsy who exhibit few seizures. During LTVEM, ASMs are gradually withdrawn to induce seizures during monitoring.^6^, ^15^

Compared with adults, the clinical characteristics of children are unique since brain development occurs during the preschool age. Children with epilepsy exhibit more frequent and diverse types of seizures (eg, age‐related epileptic spasms). These factors might also affect the choice of ASM that has to be withdrawn during LTVEM in children; however, no previous study has focused on the protocol for ASM tapering in children with epilepsy. Although the guidelines published by the ILAE in 2021 for LTVEM include ASM tapering,^6^ these guidelines are not focused on children. This study summarizes our experience of using the PUFH protocol for the rapid withdrawal of ASMs during LTVEM in the past 4 years. To the best of our knowledge, this is the first study on ASM withdrawal during LTVEM with a focus on children with epilepsy. Furthermore, the average age of subjects was much lower in our study compared to that in previous studies.^6^

Herein, using the PUFH protocol for rapid withdrawal of ASMs during LTVEM, seizures were successfully induced in 90% of children with nondaily seizures. Compared with the study by Kasab et al^8^ in which ictal EEG was obtained in 76% of patients after ASM tapering, our protocol had a higher success rate for inducing seizures. Kumar et al^10^ analyzed 70 patients and reported that the time interval between ASM tapering initiation and the first induced seizure was 2.9 ± 1.7 days, while in our study, the median duration was 3 days (2–4). Thus, our result is consistent with that of Kumar et al Furthermore, the age range of patients in the study by Kumar et al was 2‐80 years, whereas our subjects were all children, most of whom were in the preschool age group.

Rizvi et al^11^ used sleep deprivation and rapid ASM tapering simultaneously and showed that seizures can be induced in 90.5% of patients with an average time interval between ASM tapering initiation and the first induced seizure of 4.53 ± 1.44 days. In our study, using the PUFH protocol for ASM tapering, seizures were successfully induced in 72 children, and 87.5% of them responded by tapering 1–2 ASMs. For most of these 72 patients, the duration between ASM tapering initiation and the first induced seizure was <4 days, which is comparable to or shorter than that reported in previous studies. We suggest that the use of the PUFH protocol for rapid withdrawal of ASMs will further reduce the length of hospital stay and decrease the cost of treatment.

Previous studies showed that rapid ASM withdrawal affects IED sites, thus making it difficult to localize EZ.^6^, ^11^, ^15^ In our study, the location and morphology of IED in the interictal state were not affected following ASM tapering in most patients, suggesting that the epileptic network in most patients was not changed and did not interfere with the localization of EZ. In the eight patients with increased interictal IED, the newly formed IED sites were identified based on the discharge at the original site. We believe that increase in the number of IED sites will not interfere with the identification of the original EZ while comparing the IED sites identified during the first 24‐hour EEG monitoring and at the beginning of seizure onset during LTVEM. Previous studies suggest that ASM has an inhibitory effect on IED;^16^ therefore, modified IED patterns observed in our study might be due to the increase in the original IED during ASM tapering. Our results showed that the PUFH protocol for rapid withdrawal of ASMs during LTVEM had a minimal impact on IED sites, suggesting that more effective electro‐clinical information can be obtained for subsequent epilepsy surgery.

The ILAE guidelines suggest that rapid withdrawal of ASMs might result in nonhabitual seizures, cluster seizures, and SE.^6^ However, in our study, seizures induced in most of the patients were habitual seizures, and only 6 patients developed new seizure types, with rare events such as cluster seizures and SE. The incidence of SAEs in our study was very low (5%), which is different from those reported in previous studies.^10^ This difference might be because the study subjects in our study were young children. Our findings are consistent with those of two previous studies^11^, ^12^ showing that rapid ASM withdrawal is safe and effective, and could efficiently reduce the length of hospital stay. Our results also suggest that rapid ASM withdrawal is safe in younger children.

There are limitations to our study. First, since this is a single‐center retrospective study with small sample size, the reliability of our conclusions needs further verification. Second, although we employed stringent inclusion criteria, it cannot be concluded that all seizures are due to ASM tapering, and the possibility of having a spontaneous seizure onset cannot be overlooked. Finally, because of different ASMs and variability in the case number of a single ASM application, we tried to evaluate every single ASM; however, the test efficiency was very low. Therefore, we did not follow up on this work and have planned to perform a multicenter study with large samples in the future.

In conclusion, our study suggests that the PUFH protocol is safe and effective for the rapid withdrawal of ASM during LTVEM in children with DRE who exhibit nondaily seizures. By tapering one or two ASMs, it is possible to induce habitual seizures within 4 days. The ictal and interictal EEG obtained during our protocol can provide meaningful electro‐clinical information for presurgical evaluation.

## CONFLICT OF INTEREST

Neither of the authors has any conflict of interest to disclose.

## ETHICAL PUBLICATION STATEMENT

This was a retrospective cohort study, patient identity remained anonymous, and the requirement for informed consent was approved to waive by the Ethics Committee of Peking University First Hospital due to the observational nature of the study. We confirm that we have read the Journal's position on issues involved in ethical publication and affirm that this report is consistent with those guidelines.
